# Isolation of broadly neutralizing HIV-1 antibodies from high-throughput single B cell culture

**DOI:** 10.1186/1742-4690-9-S2-P91

**Published:** 2012-09-13

**Authors:** NS Longo, N Doria-Rose, K McKee, S O'Dell, MK Louder, Z Yang, R Bailer, JR Mascola

**Affiliations:** 1 NIH, Vaccine Research Center, Bethesda, MD, USA

## Background

The isolation of broadly neutralizing HIV-1 antibodies that arise during infection has provided insights into the design of vaccine immunogens capable of eliciting similar antibody response. The use of HIV-specific sorting probes resulted in the isolation of antibodies to vulnerable viral epitopes, such as the CD4-binding site, but the use of such probes does not explore the subject’s immunoglobulin repertoire breadth.

## Methods

To identify new HIV-1 monoclonal antibodies (mAbs), we developed a B-cell culture system to isolate and screen thousands of B cells. Memory B cells were isolated by negative selection and individually cultured in 384 well plates with irradiated feeder cells expressing CD40 ligand. The addition of cytokines IL-2 and IL-21 stimulated proliferation and immunoglobulin secretion.

## Results

After 14 days in culture, approximately 35% of the B cell clones secreted >100 ng/ul of IgG which met the sensitivity threshold to screen each clone in an automated micro-neutralization assay. B cell clonal expansion allowed recovery of the immunoglobulin heavy and light chains by RT-PCR, with subsequent cloning into expression vectors and mAb testing against a large panel of viruses. In one experiment screening approximately 8600 B cells, 9 clones were identified as potential contributors to the neutralization detected in the patient’s serum. One of the isolated clones produced mAb VRC22 with 30% neutralization breadth and moderate potency. Further studies revealed that VRC22 was sensitive to JRCSF glycan mutants N332A and N301A but not N160K. VRC22 utilizes VH4-34 with a single amino acid CDR1 deletion and a mutation frequency of 7% which is a lower level of affinity maturation than observed for most known HIV-1 neutralizing antibodies.

## Conclusion

This B-cell culture system allows efficient screening of thousands of individual B cells and the recovery of antigen specific mAbs. This approach can be used to isolate human mAbs to diverse pathogens.

